# Mitochondria-mediated Ferroptosis in Diseases Therapy: From Molecular Mechanisms to Implications

**DOI:** 10.14336/AD.2023.0717

**Published:** 2024-04-01

**Authors:** Fuhai Feng, Shasha He, Xiaoling Li, Jiake He, Lianxiang Luo

**Affiliations:** ^1^The First Clinical College, Guangdong Medical University, Zhanjiang, Guangdong, China.; ^2^Beijing Hospital of Traditional Chinese Medicine, Capital Medical University, Beijing, China.; ^3^Animal Experiment Center, Guangdong Medical University, Zhanjiang, China.; ^4^The Marine Biomedical Research Institute, Guangdong Medical University, Zhanjiang, Guangdong, China.; ^5^The Marine Biomedical Research Institute of Guangdong Zhanjiang, Zhanjiang, Guangdong, China.

**Keywords:** Mitochondria, ferroptosis, lipid peroxidation, iron metabolism, disease

## Abstract

Ferroptosis, a type of cell death involving iron and lipid peroxidation, has been found to be closely associated with the development of many diseases. Mitochondria are vital components of eukaryotic cells, serving important functions in energy production, cellular metabolism, and apoptosis regulation. Presently, the precise relationship between mitochondria and ferroptosis remains unclear. In this study, we aim to systematically elucidate the mechanisms via which mitochondria regulate ferroptosis from multiple perspectives to provide novel insights into mitochondrial functions in ferroptosis. Additionally, we present a comprehensive overview of how mitochondria contribute to ferroptosis in different conditions, including cancer, cardiovascular disease, inflammatory disease, mitochondrial DNA depletion syndrome, and novel coronavirus pneumonia. Gaining a comprehensive understanding of the involvement of mitochondria in ferroptosis could lead to more effective approaches for both basic cell biology studies and medical treatments.

## 1. Introduction

Cancer and cardiovascular diseases are significant global challenges, characterized by increasing mortality rates. The underlying causes often involve cell dysfunction or cell death in affected tissues and the emergence of drug-resistant tumor cells, highlighting the urgent need for new and effective treatments. Extensive research has revealed the crucial role of mitochondria in various cellular processes, including ATP production in metabolic centers via the TCA cycle, calcium stabilization and signaling, hormone production, cell growth, cell differentiation, cell cycle control and cell death, which are essential for proper cell functioning [1-4]. Thus, it is plausible to hypothesize that mitochondrial dysfunction, altered mitochondrial activity, or disruptions in mitochondria-regulated signaling pathways may contribute to the development of the aforementioned diseases [5, 6], and targeting mitochondria to modulate the function and survival of diseased cells could be a promising therapeutic approach.

The concept of ferroptosis was formally introduced in 2012 [7]. Ferroptosis is a unique form of cell death caused by iron-mediated lipid peroxidation that differs from other regulated cell death (RCD) mechanisms such as apoptosis, autophagy, necroptosis and pyroptosis in terms of its morphology and underlying mechanisms [8]. The progression and cellular sensitivity to ferroptosis are influenced by iron metabolism, energy metabolism, lipid metabolism, and specific molecules. As our understanding of ferroptosis has advanced, it has become increasingly clear that this form of cell death is closely associated with various diseases, including cancer, cardiovascular diseases, inflammatory diseases, and others [9-14]. Moreover, targeting ferroptosis as a treatment approach has shown promise in these diseases, with the development of inhibitors such as ferrostatin-1 (fer-1) and liproxstatin-1 (lip-1) [15], as well as the use of anticancer drugs like sorafenib to modulate cancer and cardiovascular diseases through RCD mechanisms.

Mitochondria, a double-membrane organelle, possess RCD capabilities that can induce cell death by releasing or attracting promoters, including ferroptosis, a newly discovered form of RCD. Recent research has demonstrated that mitochondria play a crucial role in erastin-induced or cysteine deprivation-induced (CDI) ferroptosis, while they are not necessary for RSL3-induced ferroptosis [16], which confirms that cells lacking mitochondria exhibit resistance to erastin, although high concentrations of erastin can still lead to cell death [17]. Erastin targets voltage-dependent anion channels (VDACs) to regulate the production of reactive oxygen species (ROS) within cells, thereby exerting its anticancer effects [17, 18]. Additionally, specific techniques that eliminate oxidation-related substances or iron in cardiomyocytes and neuronal cells have demonstrated the ability to block mitochondria-mediated ferroptosis [19-23]. Numerous studies have confirmed the critical role of mitochondria in the occurrence of ferroptosis, emphasizing their tremendous significance. Therefore, targeted approaches aimed at removing oxidation-related substances or iron from cardiomyocytes and neuronal cells hold potential for inhibiting the mitochondria-mediated pathways of ferroptosis.

The connection between mitochondrial function and ferroptosis has been a topic of debate, as various studies have shown that susceptibility to ferroptosis is not correlated with mitochondrial DNA (mtDNA) deletion or the removal of mitochondria, which presents a paradoxical situation [7, 24]. However, recent research on the mitochondrial NEET protein, an iron-sulfur protein (2Fe-2S), has highlighted the importance of mitochondrial iron metabolism in the context of ferroptosis, as it plays a critical role in maintaining cellular iron and ROS homeostasis [25]. Furthermore, additional studies have provided supporting evidence for the involvement of mitochondria in ferroptosis [26, 27]. Despite these findings, a consensus has not yet been reached in this field, and there is a limited amount of literature available on the subject. To address this gap, this review focuses on the morphological, metabolic, and molecular changes in mitochondria during ferroptosis. It explores the intricate interplay between ROS, iron regulation, energy metabolism, and other crucial molecules associated with ferroptosis. The review also examines the role of mitochondria-mediated ferroptosis in various diseases and discusses the typical molecules involved in mitochondria-mediated ferroptosis as potential targets for disease treatment. To gain a deeper understanding of the molecular processes underlying ferroptosis, investigate the regulatory mechanisms of intracellular iron metabolism and transport, and develop more effective therapies for ferroptosis, a comprehensive investigation into mitochondria-mediated ferroptosis is essential. Overall, this review summarizes recent advances in mitochondria-mediated ferroptosis, suggesting new directions for targeting mitochondria to regulate ferroptosis and offering a distinct approach to treating the diseases discussed.

## 2. Mechanism of Ferroptosis

Ferroptosis is distinctive in several aspects as a unique form of cell death that arises from specific mutations in tumor genes and the buildup of iron ions, resulting in lipid peroxidation primarily affecting mitochondria. The glutathione (GSH)-glutathione peroxidase 4 (GPX4) axis, mTOR pathway and HSP90 have been shown to significantly influence ferroptosis. By regulating the redox balance and maintaining intracellular antioxidants, it becomes possible to effectively reduce the occurrence of ferroptosis, offering hope for developing novel therapeutic approaches.

### 2.1. The Characteristics of Ferroptosis

Erastin was discovered by Dr. Stockwell in 2003 through high-throughput screening of synthetically hazardous compounds. It induces a distinct form of cell death different from apoptosis and exhibits high efficacy against cancers harboring mutant Ras. Genetically modified tumorigenic cells display significant lethal cytotoxicity when exposed to erastin, whereas their normal isogenic counterparts do not activate this response [10]. Marcus Conrad's discovery in 2008 revealed that non-apoptotic cell death could be triggered by genetic regulation of crucial genes governing the redox state [28, 29], which led to the understanding that erastin is regulated in an iron-dependent manner [30] and is highly regulated by molecular perturbations [16]. Ferroptosis is a novel type of RCD that differs from existing RCD types and other types of cell death because it can cause noticeable morphological alterations in mitochondria. Mutations in the oncogenes HRAS, KRAS, or BRAF are necessary for ferroptosis-induced cell death, which can be triggered by lethal iron-dependent lipid peroxidation and can be activated by specific small compounds such as erastin, RSL3, and sorafenib [7, 31, 32].

Biochemically, elevated levels of iron lead to the accumulation of lipid metabolites, a decrease in GPX4 activity, and a substantial increase in ROS[33]. Notably, apoptosis or necroptosis inhibitors have no effect on this type of cell death. However, using specialized techniques to eliminate intracellular iron and reduce ROS levels, it is possible to rescue the cells, indicating that ferroptosis is dependent on both iron and oxidation [34].

### 2.2. The Defense Mechanism of Ferroptosis

In contrast to other forms of RCD, ferroptosis is distinguished by its dependence on iron and its association with lipid peroxidation, which occurs through unique intracellular signaling pathways. Evidence supporting the distinctiveness of ferroptosis has continued to accumulate since its identification as a separate form of cell death [7]. The primary drivers of ferroptosis are inadequate clearance and excessive synthesis of lipid peroxides, which can arise through enzymatic or non-enzymatic mechanisms [35]. Recent advancements have shed light on enzymatic and non-enzymatic antioxidant defense strategies against ferroptosis [36]. In this context, the GSH-GPX4 axis, represented by the system xc, has been shown to play a critical role in ferroptosis. Inhibitors targeting this axis, including well-known ferroptosis inducers like erastin, RSL3, and the anticancer drug sorafenib, disrupt the uptake of cystine into cells, thereby impeding GSH production, which is vital for cellular antioxidant defenses [37]. Depletion of GSH, in turn, leads to the accumulation of ROS, causing damage to proteins and membranes and ultimately culminating in ferroptosis [38]. The antioxidant enzyme GPX4 can convert phospholipid hydroperoxides (PLOOHs) to less harmful phospholipid alcohols (PLOHs) [39]. Furthermore, GSH is a cofactor for various antioxidant enzymes, influencing the regulation of LOX activity, intracellular translocation [40, 41], as well as the redox cycling activity of Fe2+ involved in the Fenton reaction [42, 43].

During ferroptosis, the activity of SLC7A11 and GPX4 is regulated through various mechanisms. For instance, SLC7A11-mediated cystine absorption leads to increased expression of GPX4, which activates the mTOR (mechanistic targets of rapamycin) pathway [44]. In addition, GPX4 can be destabilized by heat shock protein 90 (HSP90) in some conditions [45], while it can be stabilized by heat shock protein family A (Hsp70) member 5 (HSPA5) [46]. The transcription of SLC7A11 is upregulated by nuclear factor erythrocyte 2-like 2 (NFE2L2, more commonly known as NRF2) [47] and tumor protein p53 (TP53) [48]. Recent discoveries of multiple GPX4 or SLC7A11-binding proteins have also been shown to further contribute to the regulation of this pathway during ferroptosis [49-52]. Several intracellular antioxidants, including GSH, coenzyme Q10 (CoQ10), tetrahydrobiopterin (BH4), and dopamine, collaborate to prevent lipid peroxidation in ferroptosis. Recent research has linked the flavoprotein apoptosis-inducing factor mitochondria associated 2 (AIFM2) to inhibiting ferroptosis and preserving coenzyme Q (CoQ)'s antioxidant activity in the cellular membrane, with this protein now recognized as ferroptosis suppressor protein 1 (FSP1) [53, 54]. In the absence of GSH, FSP1 can act as a substitute for GSH, enabling the normal functioning of the ferroptosis regulatory axis and preventing the formation of lipid peroxides [19]. In addition, ESCRT-III complexes, composed of CHMP5 and CHMP6, participate in the membrane repair process involved in AIFM2-mediated ferroptosis inhibition [55]. FSP1 [56] and dihydroorotate dehydrogenase (DHODH) [57] can restrict ferroptosis mechanistically by reducing ubiquinol production from the cytosol and mitochondria. GTP cyclohydrolase 1 (GCH1) also contributes to suppressing ferroptosis [58, 59]. During ferroptosis, the lipid peroxidation rate is lowered due to increased GPX4 protein stability by dopamine [60]. Furthermore, by inhibiting the hydrolysis of oxidized phosphatidylethanolamine, phospholipase A2 VI group member 6 (PLA2G6, also known as iPLA2) inhibits ferroptosis [61, 62]. Figure 2 illustrates the intracellular defensive systems that prevent ferroptosis. Collectively, these findings support the concept that a well-functioning integrated antioxidant system can protect against ferroptosis induced by oxidative stress.

## 3. The Relationship Between Mitochondria and Ferroptosis

Mitochondria play a significant role in regulating ferroptosis, as their metabolic activity is closely intertwined with this process. They serve as a major source of intracellular ROS and are involved in maintaining iron homeostasis. Various mitochondrial proteins have been identified to have regulatory roles in ferroptosis. By modulating iron homeostasis, ROS production and lipid peroxidation, mitochondria can substantially influence the regulation of ferroptosis. Therefore, understanding the mitochondrial factors that govern ferroptosis can provide valuable insights into its underlying mechanisms. Thus, this section discusses the interplay between mitochondria and ferroptosis, focusing on amino acid metabolism, lipid metabolism, iron metabolism, energy metabolism, molecular mechanisms, mitochondria-specific defense mechanisms, and unique mitophagy processes.

### 3.1. Mitochondrial Morphological Alteration and Ferroptosis

One of the most distinct characteristics of ferroptosis compared to other forms of RCD is the alteration in mitochondrial morphology. This dynamic process is driven by oxidative stress, which induces changes in the structure of both the outer mitochondrial membrane (OMM) and the cristae-structured inner mitochondrial membrane (IMM). Mitochondria are highly active organelles closely associated with mitochondrial dysfunction, energy production and maintenance of their structural integrity, which is crucial for ATP depletion during mitochondrial-cytoplasmic material transport [63, 64]. VDACs, ubiquitous proteins in the OMM, facilitate the passive transport of anionic hydrophilic metabolites, including adenosine diphosphate (ADP), adenosine triphosphate (ATP), phosphate (Pi), and respiratory substrates [65, 66]. Studies have revealed that unbound intracellular tubulin regulates VDAC, and its partial inhibition helps to restrict mitochondrial metabolism and decrease mitochondrial membrane potential [67]. Erastin and its analogs, which target mitochondrial VDAC, interfere with the effects of free tubulin on VDAC, leading to VDAC opening, increased mitochondrial membrane potential, and subsequent ROS production. Consequently, cells with higher levels of VDAC proteins are more susceptible to the mitochondrial depolarizing effect of erastin [32, 68]. Moreover, mitochondrial depolarization marks the onset of mitochondrial dysfunction and loss of mitochondrial membrane integrity [69], and variations in the fluidity of the mitochondrial membrane, caused by lipid peroxidation products, also play a crucial role in mitochondrial membrane dynamics during ferroptosis.

### 3.2. Mitochondrial Amino Acid Metabolism and Ferroptosis

**Figure 1.**
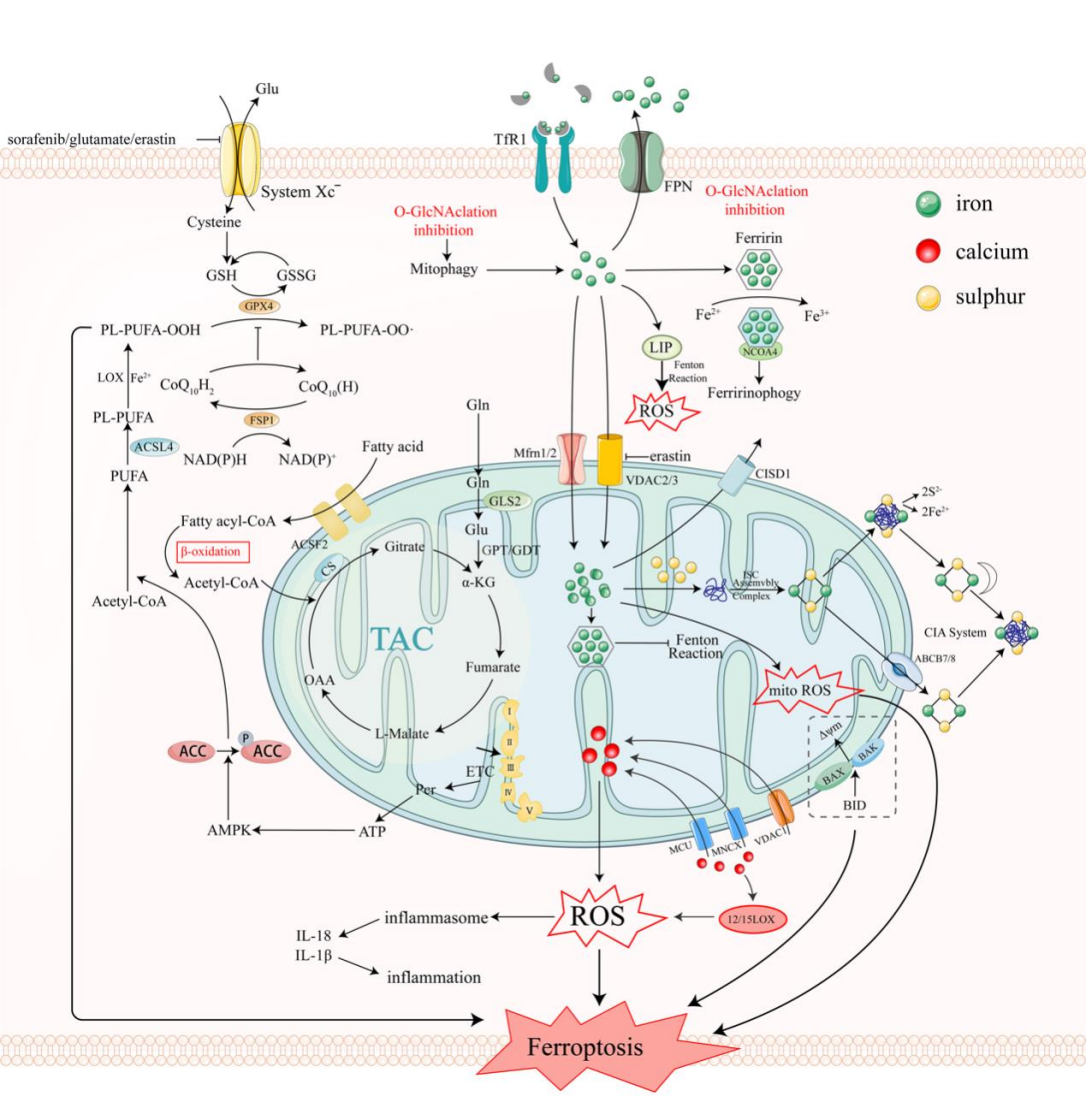
**The fundamental ferroptosis-related intracellular pathways and mitochondrial regulation**. Metabolic pathways for lipids: Xc mediates the exchange of cystine and Glu, while cystine stimulates GSH synthesis. Gln is converted to Glu, which is crucial for CDI ferroptosis, with the assistance of GIS2 and the action of GPT and GDT. ACSL4 and LPCAT 3 esterify PUFA to PE, preparing for subsequent peroxidation reactions catalyzed by LOX or POR enzymes or mediated by cellular free iron via the Fenton reaction. Iron metabolic pathway: Iron binds to TF, enters the cell via TfR1, and is then released into the LIP in the cytoplasm, where it participates in the Fenton reaction, producing ROS and promoting ferroptosis. ROS can also trigger the secretion of IL-1 and IL-18 via inflammasome signaling, contributing to inflammation. ETC stimulates ATP synthesis via PCr and activates AMPK, which inhibits ACC and decreases PUFA synthesis. Iron can enter the mitochondria through VDAC2/3, Mfrn1/2 or via the Fenton reaction, resulting in ferroptosis. Mitochondrial iron can also enter the ISC assembly machinery, which is responsible for the maturation of all cellular (Fe-S) clusters. NEET iron-sulfur proteins transfer their 2Fe-2S clusters to the apolipoprotein receptor proteins and the CIA system. FtMt is involved in mitochondrial iron storage. BID induces ferroptosis by activating BAX and BAK And CISD1 modulates iron export in mitochondria. Abbreviations: AOA, amino-oxyacetic acid; ACSF2, acyl-CoA synthetase family member 2; Glu, glutamate; Gln, glutamine; AMPK, Adenosine monophosphate-activated protein kinase; GSSG, glutathione disulfide; GSH, glutathione; PUFA, polyunsaturated fatty acid; GPT, glutamate-pyruvate transaminase; ACSL4, *acyl-CoA synthetase long-chain family* member 4; TfR1, transferrin Receptor 1; GLS2, glutaminase 2; Mfrn1/2, mitoferrin 1/2; mitoROS, mitochondrial ROS; LIP, labile iron pool; BID, BH3 interacting-domain death agonist; BAK, Bcl-2 homologous antagonist killer; VDAC2/3, voltage-dependent anion channels 2/3; BAX, Bcl-2-associated X protein; ETC, electron transport chain; CISD1, CDGSH Iron Sulfur Domain 1; FtMt, mitochondrial ferritin; ACSL4, acyl-CoA synthetase long-chain family member 4; MCU, mitochondrial calcium uniporter; LOX, lipoxygenase; CoA, coenzyme A; α-KG: alpha-ketoglutarate; GDH, glutamate dehydrogenase; GOT, glutamic oxaloacetic transaminase; TCA cycle, tricarboxylic acid cycle; PCr, phosphocreatine; ATP, adenosine triphosphate; ROS, ROS; IL-1β, Interleukin-1β; 12/15LOX, 12/15 lipoxygenase; FPN, ferroportin; IL-18, Interleukin-18; ACC, acetyl-CoA carboxylase; MNCX, mitochondrial sodium calcium exchanger.

Amino acid metabolism is intricately connected to ferroptosis, and mitochondria play a pivotal role in both processes. Specifically, two members of the protein complex system xc, SLC7A11 and SLC3A2, are responsible for amino acid transport [70]. Among these amino acids, cystine, the disulfide form of cysteine, is particularly important for promoting the synthesis of GSH. System xc facilitates the exchange of cystine and glutamate within the organism. GPX4 is an essential enzyme involved in the elimination of lipid peroxides. When system xc is inhibited, the levels of GSH are reduced, and the action of GPX4 is hindered, rendering cells more susceptible to ferroptosis. Cysteine, a key component of GSH, plays a critical role in maintaining stable cellular redox levels, which is crucial for the antioxidant activity of the intracellular GSH-GPX4 axis. Furthermore, cysteine contributes to GSH production by providing sulfur for forming Fe-S clusters through the cysteine desulfurase complex NFS1-ISD11 (Figure 1) [71]. Cysteine depletion has been shown to cause bone hyperplasia in cancer [72], and mitochondria have been implicated in CDI ferroptosis.

The non-essential amino acid glutamine (GLN) is degraded by the enzyme glutaminase (GLS) degrades the non-essential amino acid GLN within mitochondria. GLN is a crucial respiratory substrate for energy production and lipid synthesis in various cells, including tumor cells. The breakdown of GLN plays a significant role in the regulation of ferritin synthesis, and its metabolites provide precursors for the TCA cycle, which is particularly important in metabolically demanding conditions[73], such as pancreatic cancer, glioblastoma multiforme, acute myeloid leukemia, and certain lung cancers [74]. In CDI ferroptosis, the downstream metabolites of the TCA cycle, specifically α-ketoglutarate (-KG) and succinate, can mimic the effects of GLN [20]. Cysteine deprivation or erastin treatment alone cannot induce mitochondrial breakdown and cell death in the absence of GLN. Inhibiting the conversion of GLN to α-KG using aminooxy acetic acid (AOA) consistently rescues cells from CDI ferroptosis [7, 9]. The degradation of GLN is mediated by two mitochondrial enzymes, GLS1 and GLS2. Genetic or pharmacological reduction of GLS2, but not GLS1, inhibits cell-associated ferroptosis (Figure 1) [9]. Despite their structural and kinetic differences, these two isoenzymes share similar amino acid sequences encoded by unrelated genes [75]. These observations may provide insights into the distinct functions of the two glutaminase classes during ferroptosis.

### 3.3. Mitochondrial Lipid Metabolism and Ferroptosis

Mitochondria are widely recognized as semi-autonomous organelles comprising two membranes, split into OMM and IMM [76, 77], that play a crucial role in lipid metabolism. Lipid metabolism in mitochondria is facilitated by phospholipids (PL) and other amphiphilic molecules, rendering mitochondria as the most prominent intracellular organelle involved in lipid metabolism. Various mitochondrial enzyme systems are involved in lipid metabolism, including acyl-CoA synthetase long-chain family member 4 (ACSL4), lysophos-phatidylcholine acyltransferase 3 (LPCAT3), and acyl-CoA synthetase family member 2 (ACSF2). These enzymes are indispensable for the occurrence of ferroptosis. ACSF2, localized in mitochondria, regulates the activation and synthesis of fatty acids, which is essential for lipid peroxidation and is believed to play a critical role in erastin-induced ferroptosis [7]. One pivotal step in fatty acid metabolism is fatty acid beta-oxidation (FAO), which involves the sequential removal of two carbon units from the acyl chain through a series of enzymatic reactions within the mitochondrial matrix, where multiple enzymes work together to form an FAO system [78]. Dysregulation of FAO is observed in many human malignancies, and targeting this pathway holds promise for developing advanced therapies to combat cancer progression, survival, stemness, drug resistance, and metastasis [79].

The endoplasmic reticulum (ER) and mitochondrial outer membrane matrix (OMM) each contain five unique isoforms of long-chain acyl-CoA synthetase (ACSL). ACSL plays a crucial role in lipid metabolism[80, 81], which indicates they may be indirectly associated with ferroptosis by participating in the synthesis of an intermediate product known as acyl-CoA and catalyzing lipid synthesis and fatty acid degradation in vivo. Among the ACSL isoforms, ACSL4 is a significant pro-ferroptosis gene in OMM. Long-chain polyunsaturated fatty acids (PUFAs) are covalently associated with ACSL4 via coenzyme A, and ACSL4 prefers arachidonic acid (AA), including AdA, as a substrate. Subsequently, AA is converted to PL-PUFA via the esterification pathway catalyzed by LPCAT3This item is converted to PL-PUFA via the LPCAT3[53, 82, 83] esterification pathway, which is subsequently peroxidized by the iron Fenton reaction or LOX[34, 84]. As a driver of ferroptosis, ACSL4 can also be used as a reference indicator of ferroptosis sensitivity [85], and breast cancer cell lines with preferential expression of ACSL4 have demonstrated increased susceptibility to ferroptosis [53]. In addition, pharmacological inhibition of ACSL4, such as TZD, holds promise as a potential intervention for ferroptosis [53]. Furthermore, the E-cadherin-mediated Merlin-Hippo-YAP pathway regulates the expression of ACSL4, influencing both cell-cell contact and ferroptosis sensitivity [86], and ACSL4 has been identified as a critical regulator of ferroptosis through this signaling pathway, suggesting its essential role in the process [87]. Additionally, the PRDX2-MFN2-ACSL4 pathway has been shown to be of utmost importance in mitochondria-associated ferroptosis, particularly in protecting diabetic heart microvasculature [88]. The relationship between mitochondria and lipid-related disorders (as shown in Figure 1) may be partially explained by the downregulation of ACSL4 expression in response to mitochondrial damage.

Acyl-CoA synthetase long-chain family member 1 (ACSL1) is required to promote ferroptosis when conjugated linolenic acid, such as alpha-eleostearic acid, is produced by certain plants [89]. On the other hand, ACSL3 is necessary for the anti-ferroptotic actions of monounsaturated fatty acids (MUFAs) like oleic acid [90]. Another mitochondrial enzyme, acyl-CoA synthetase long-chain family member 5 (ACSL5), is responsible for converting free long-chain fatty acids with 16-18 carbons, including oleic acid, linoleic acid, and palmitic acid, into fatty acyl-coenzyme A. ACSL5 has been implicated in pro-apoptosis and tumor growth prevention in cancer [80]. However, the specific role of ACSL5 in ferroptosis is yet to be established, necessitating further research on this topic. Taken together, ferroptosis, which can significantly impact various diseases, is regulated by a cascade of mitochondrial components involved in lipid synthesis and the control of lipid oxidation processes.

### 3.4. Mitochondrial Iron Metabolism and Ferroptosis

Mitochondria, organelles characterized by double membranes (OMM and IMM with cristae structure), contain a labile iron pool (LIP) that exhibits strong redox activity [20]. This iron pool is crucial to metabolize ROS mentioned earlier. Iron, the most abundant metal in mitochondria, is vital in numerous physiological functions. It is involved in the synthesis of iron-sulfur clusters (Fe-S), heme, and other cofactors, which are essential for various cellular processes (Figure 1) [42, 91, 92]. However, the precise mechanism by which iron contributes to mitochondria-induced ferroptosis remains unknown.

Iron must traverse both its OMM and IMM to be metabolized within the mitochondrial matrix. Mitochondria have specific mechanisms for iron entry, including the involvement of the mitoferritin transporters Mfrn1/2 [21]. In recent years, ferroptosis has been implicated in various neurological diseases[23], including Alzheimer's, Huntington's, Friedreich's ataxia and Parkinson's. Iron is transported across the gap between IMM and OMM through the VDACs in the OMMs and into the mitochondrial matrix [93]. Notably, erastin can induce mitochondria-mediated ferroptosis by binding to VDAC2/3 [18]. Additionally, oligomerization of VDAC1 can also impact the potential and permeability of mitochondrial membranes, facilitating intracellular iron transfer to the Fenton enzyme and subsequent initiation of oxidative damage, lipid peroxidation, and ferroptosis [94]. Components of the electron transport chain (ETC), including Fe-S cluster-containing proteins and heme-containing proteins in the IMM complex, heavily rely on mitochondrial matrix iron as a cofactor [95]. Increased mitochondrial protein frataxin (FXN) expression provides cellular protection against iron deficiency and erastin-induced ferroptosis (Figure 1). FXN is a highly conserved protein that functions as a molecular chaperone of iron and activates the driving Fe-S cluster in the mitochondria. In addition, decreased expression of FXN leads to elevated iron accumulation within mitochondria, resulting in mitochondrial dysfunction, altered morphology, increased lipid peroxidation, and susceptibility to ferroptosis[96].

Mitochondrial ferritin (FtMt) plays a dual role in lipid peroxide production. On the one hand, FtMt inhibits the accumulation of mitochondrial ROS [97], protecting mitochondrial structures and genetic material from damage and ensuring the proper functioning of essential biological reactions, which also helps maintain ATP production and prevents energy deficiency and cell death[98]. On the other hand, FtMt regulates the availability of free iron in the body [99], thereby preventing iron aggregation, oxidative stress, and ferroptosis in conditions such as brain I/R injury and indicating that FtMt could be considered a potential target for preventing ferroptosis-induced brain injury [100]. Similar to cytoplasmic ferritin in structure and iron oxidase and iron-binding properties, FtMt is a recombinant heavy polypeptide of ferritin[101]. Reduced expression of FtMt leads to an increase in free iron levels, which can participate in the Fenton reaction, generating ROS and ultimately contributing to ferroptosis (Figure 1) [102]. The exchange of calcium between the endoplasmic reticulum (ER) and mitochondria has been observed through studies of mitochondria-associated membranes (MAMs)[103]. However, the exchange of mitochondrial iron with iron in other organelle membranes or cell membranes and the extent to which mitochondria independently metabolize iron in relation to other organelles or cellular iron metabolism remains debatable [104].

NEET proteins, a class of iron-sulfur proteins, have emerged as significant players in clinical research due to their involvement in regulating iron and ROS in cancer cells and their potential role in promoting tumor cell survival [25]. These NEET protein clusters are found to be highly expressed in various cancer types, including within mitochondria. Deletion of the mitoNEET protein disrupts iron homeostasis in the striatum of knockout mice, leading to increased levels of ROS and subsequent striatal dysfunction (Figure 1). The CDGSH iron-sulfur domain-containing protein 1, also known as mitoNEET, plays an important role in maintaining normal mitochondrial function and intra-mitochondrial iron balance [105]. The relationship between Parkinson's disease (PD) and mitoNEET is closely intertwined, and understanding their interplay may shed light on PD pathology [106].

### 3.5. Mitochondrial Energetic Metabolism and Ferroptosis

Mitochondria, referred to as intracellular powerhouses, are not only the main producers of intracellular ROS and primary sites for iron utilization but also provide cells with a substantial energy source via two well-known metabolic pathways: oxidative phosphorylation (OXPHOS) and TCA [107]. To back up their beneficial involvement in promoting ferroptosis, TCA and OXPHOS positively affect the buildup of ROS in mitochondria[108]. One important metabolite in the TCA cycle, α-ketoglutarate (α-KG), is essential for lipid peroxidation [9]. In instances of high metabolic demand, such as in pancreatic cancer, glioma multiforme, acute myeloid leukemia and small-cell lung cancer, GLN serve as a precursor for the TCA cycle, where the enzyme that catalyzes GLN catabolism is also found in the mitochondria. Moreover, under conditions of GSH depletion, ETC-mediated ROS production is indispensable for glutamate and cystine deprivation-induced cell death[20, 40, 109]. Mitochondrial depletion has been found to increase tolerance to CDI ferroptosis. Furthermore, inhibiting canonical metabolic activities, such as mitochondrial TCA and ETC, contributes to inhibiting CDI ferroptosis (Figure 1) [110]. Enzymatic reactions such as α-KG dehydrogenase and α-glycerophosphate dehydrogenase[111], monoamine oxidase, nicotinamide adenine dinucleotide phosphate (NADPH) oxidase 4 and monoamine oxidase can also generate ROS in the mitochondria[112] and play a role in increased ROS production and RSL3-induced mitochondrial ferroptosis [20]. NADPH, a product of the TCA cycle, not only contributes to energy production through coupling with OXPHOS but also acts as a crucial intracellular reducing agent required for the elimination of lipid hydroperoxides, thereby affecting ferroptosis sensitivity. Measurement of NADPH levels can serve as an indicator of ferroptosis sensitivity in various cancer cell lines [113].

The electron transport of the ETC complex, which operates in conjunction with ATP synthase and uses CoQ as an electron carrier [114, 115], is responsible for energy production and generates proton power. Recent studies have demonstrated that AMP-activated protein kinase (AMPK), a central regulator of cellular energy balance, is activated in response to energy depletion, such as glucose deprivation, and that AMPK inactivation increases ferroptosis [116, 117]. ETC can also stimulate ATP production [118] via phosphocreatine (PCr), thereby activating AMPK, which inhibits acetyl-CoA carboxylase (ACC) and ferroptosis (Figure 1) [116].

### 3.6. Mitochondrial Other Molecules and Ferroptosis

FUN14 domain-containing 2 (FUNDC2) is a mitochondrial protein that has been reported to regulate mitochondria-dependent platelet death in oxygen-deprived conditions. FUNDC2 is an extra-compartmental membrane protein with two transmembrane domains. In the context of doxorubicin (DOX)-induced cardiomyopathy, FUNDC2 has been shown to interact with SLC25A11, leading to decreased stability of SLC25A11 and downregulation of mitochondrial GSH, ultimately promoting ferroptosis [20, 119]. Considering that SLC25A11 is responsible for transporting GSH into the mitochondrial matrix and FUNDC2 can impact the stability of GPX4 (Figure 1), exploring the influence of FUNDC2 on other oxidation pathways or its interaction with other GSH transporters could provide further insights into the role of the FUNDC2-SLC25A11 axis in mitochondria-mediated ferroptosis [120].

**Table 1:** Ferroptosis and mitochondria-related molecules.

Classification	Molecule	Name	Mechanism	Reference
**Pro-ferroptosis factors**	ACSF2	Acyl-CoA syntheses family member 2	Regulating intracellular fatty acid metabolism by catalyzing long-chain fatty acid and longer acyl-CoA combinations.	[7]
	FUNDC2	FUN14 domain-containing 2	lowering mitoGSH by inhibiting SLC25A11 stability and dimer formation and triggering ferroptosis.	[120]
	VDAC1	voltage-dependent anion channels 1	VDAC1 oligomerization causes abnormal iron metabolism, mitochondrial damage, and ferroptosis in hepatocytes	[94]
	FtMt	Mitochondrial ferritin	Stores free iron in mitochondria	[97]
	ACSL4	*Acyl-CoA synthetase long-chain family* member 4	Catalyzes several PUFAs, participates in fatty acid metabolism and contributes to the level of lipid peroxidation	[85]
	CS	*citrate synthase*	Contributing to lipid synthesis provides precursors for TCA and induces ferroptosis.	[7]
**Anti-ferroptosis factors**	DHODH	Dihydroorotate dehydrogenase	CoQ is converted to the antioxidant CoQH2 by a reduction process, protecting cells from ferroptosis.	[57]
	CISD1	CDGSH iron sulfur domain 1	Reducing mitochondrial iron accumulation to resist ferroptosis	[105]
	FXN	frataxin	Essential for iron-sulfur biosynthesis	[137]
	mGPX4	mitochondrial glutathione peroxidase 4	Preventing mitochondrial GSH oxidation to resist ferroptosis	[138]
	GPD2	G3P dehydrogenase 2	Converting G3P oxidation to DAP and Reducing CoQ to CoQH2 to resist ferroptosis	[139]
	STARD7	STAR-related lipid transfer domain containing 7	cytoplasmic STARD7 transfers CoQ, eliminating the function of plasma membrane lipid peroxidation	[140]

Under physiological conditions, several dehydrogenases involved in the TCA cycle are Ca2+-dependent, indicating that mitochondrial metabolism is influenced by the absorption of mitochondrial Ca2+ ions [121-123]. Elevated cytosolic calcium levels have been found to activate 12/15-lipoxygenase (12/15LOX)[124], which in turn affects ROS production. The increase in intracellular Ca2+ and excessive ROS generated during oxidation lead to disturbances in mitochondrial membrane potential and overall mitochondrial dysfunction due to mitochondrial Ca2+ overload [125-127]. Thus, maintaining mitochondrial homeostasis relies on the regulation of Ca2+ influx and efflux through various channel proteins. Mitochondrial Ca2+ uptake is facilitated by mitochondrial Ca2+ uniporters (MCUs), mitochondrial Na+/Ca2+ exchangers (mNCX), and mitochondrial H+/Ca2+ exchangers (HCX) [126, 128-130]. Inhibiting mitochondrial Ca2+ uptake using nelfinavir or the MCU inhibitor Ruthenium Red can protect mitochondria from injury and oxidative stress [131]. DOX, a chemotherapeutic drug, has been associated with various adverse cardiac effects, including iron-mediated ROS production, mitochondrial dysfunction, respiratory disruption, and alterations in reticulum Ca2+ flux (Figure 1)[132]. Calcium chelators have been shown to inhibit ferroptosis in LUHMES cells [133]. Additionally, restricting cellular Ca2+ influx has been found to protect against ferroptosis [134]. Thus, maintaining adequate Ca2+ ion balance within mitochondria is crucial for mitigating mitochondria-mediated ferroptosis to ensure the maintenance of mitochondrial structure, metabolism and redox balance, and targeting Ca2+ levels could be an innovative approach for inhibiting ferroptosis via mitochondrial interventions.

The pro-apoptotic Bcl-2 family member BH3 interacting-domain death agonist(BID) is cleaved by caspase-8 and translocated to mitochondria as a caspase-mediated apoptosis signal[135]. Recent research has revealed the significant impact of BID on mitochondrial morphology and function during neuronal ferroptosis. Inhibition of BID has been shown to suppress erastin-induced ferroptosis, while ferroptosis inhibitors protect cells from mitochondrial dysfunction and ferroptosis [136]. However, previous studies have demonstrated that inhibitors of apoptosis have limited efficacy in modulating BID's effects. In the glutamate-induced oxytocin model, BID has been found to enhance mitochondrial ROS generation and disrupt mitochondrial membrane integrity, as evidenced by changes in mitochondrial membrane potential (MMP) and the release of apoptosis-inducing substances [127]. A recent study [27] showed that knocking down BID or using a BID inhibitor can prevent the onset of ferroptosis. As shown in Figure 1, BID induces ferroptosis by activating the pro-apoptotic proteins Bcl-2-associated X protein (BAX) and Bcl-2 homologous antagonist killer (BAK), leading to dysregulation of mitochondrial membrane potential. Additionally, erastin- or RSL3-induced ferroptosis heavily relies on the presence of BAX and BAK [16]. BID can modulate the potential and permeability of mitochondrial membranes, allowing the influx of iron ions and initiating damaging reactions such as oxidative stress and lipid peroxidation, ultimately resulting in ferroptosis. It also plays a crucial role in neuronal ferroptosis, and inhibiting its oligomerization or reducing its activity might prevent or be used as a strategy to treat neurological disorders associated with ferroptosis [136]. These reports shed light on the involvement of mitochondrial damage in ferroptosis. Table 1 provides an overview of chemicals targeting mitochondria that have been shown to either promote or prevent ferroptosis.

### 3.7. Mitochondrial Defense Mechanisms and Ferroptosis

Considering the defense systems present in the cytoplasm and other cell membranes, such as cytoplasmic GSH peroxidase 4 (GPX4) and ferroptosis suppressor protein 1 (FSP1), are unable to enter the mitochondria and remove the accumulated lipid peroxides in the inner mitochondrial membrane, mitochondria are particularly susceptible to lipid peroxidation, leading to compromised membrane integrity and dysfunction [141].

Ferroptosis sensitivity was observed to be independent of mtDNA deletion or mitochondrial removal[7, 24], and GPX4 inactivation-induced ferroptosis did not result in significant mitochondrial lipid peroxidation, suggesting that mitochondria may not play a prominent role in ferroptosis [142]. Although there are both cytoplasmic and mitochondrial forms of GPX4, studies have indicated that cytoplasmic GPX4 is essential for rodent embryonic development rather than mitochondrial GPX4. In addition, it is believed that cytosolic GPX4, not mitochondrial GPX4, plays a role in the protection against ferroptosis [143-146]. Notably, activation of mitochondrial GPX4 has been shown to inhibit ferroptosis. Overexpression of mitochondrial GPX4 has been demonstrated to protect cells against ferroptosis induced by DOX[138], indicating a potential function of mitochondrial GPX4 in mitochondria.

DHODH is an enzyme located in IMM that can reduce CoQ to CoQH2, thereby neutralizing peroxyl radicals in mitochondrial lipids and preventing ferroptosis [115, 147]. The presence of DHODH in mitochondria and its ability to neutralize lipid peroxides support its protective role against ferroptosis, and the absence of DHODH has been shown to induce ferroptosis [57]. Thus, the interference of DHODH with ferroptosis has emerged as a prospective target for treating various diseases [57, 148, 149].

Recent research has provided further evidence of mitochondrial resistance against the ferroptosis system. Metabolomic studies have revealed that glycerol (G3P) is downregulated in cells following treatment with a GPX4 inhibitor. Further investigation identified G3P dehydrogenase 2 (GPD2), an enzyme located in the IMM, as an inhibitor of ferroptosis. GPD2 functions in a CoQ-dependent manner by converting G3P oxidation to dihydroxyacetone phosphate (DAP), transferring electrons to the ETC in the mitochondria and reducing CoQ to CoQH2. Inhibition of GPX4 leads to mitochondrial lipid peroxidation and ferroptosis in cancer cells with a deficiency of GPD2. FSP1 and DHODH, by generating CoQH2 in the plasma membrane and mitochondria, also suppress ferroptosis. Overexpression of DHODH, but not FSP1, significantly rescues the ferroptosis-sensitive phenotype of GPD2-deficient cells. Similarly, overexpression of GPD2, which works alongside DHODH in mitochondria to prevent ferroptosis, partially rescues the phenotype of DHODH-deficient cells. However, this effect is not observed at the plasma membrane, where FSP1 is present. Notably, the tumor-suppressing effects of GPD2 and mitochondrial GPX4 against ferroptosis are additive, highlighting the contribution of GPD2 in mitochondrial ferroptosis resistance [139].

**Figure 2.**
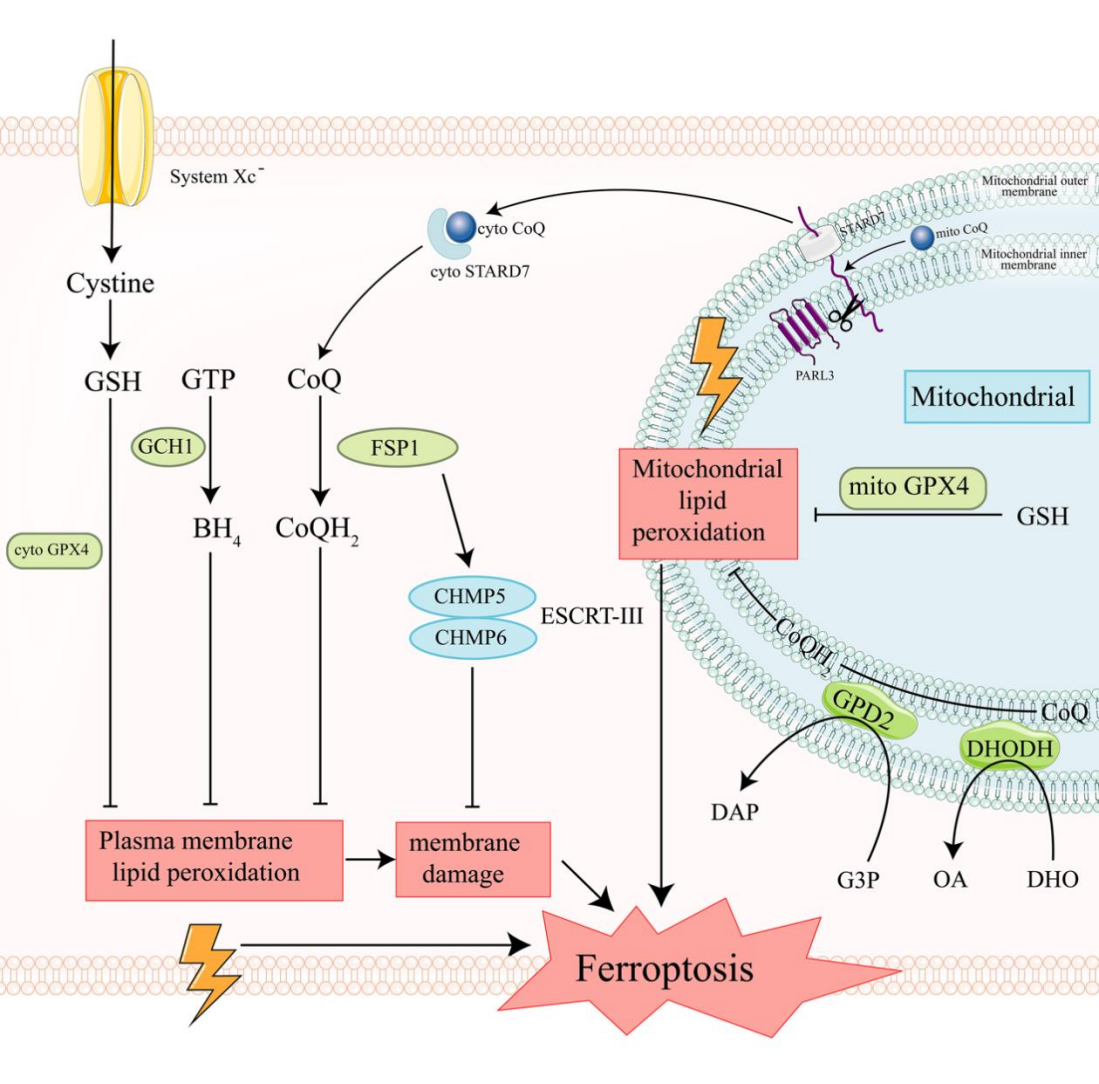
**Ferroptosis defensive systems inside cells and mitochondria**. In the cytoplasm and mitochondria, cells have developed seven defense mechanisms to prevent ferroptosis. In the cytoplasm, they are GPX4, FSP1 and GCH1, and in the mitochondria, they are GPX4, DHODH and GPD2. STARD7 is present in both the cytoplasm and mitochondria. Abbreviation used: GSH, glutathione; STARD7, StAR-related lipid transfer domain containing 7; GPX4, glutathione peroxidase 4; BH4, tetrahydrobiopterin; cyto, cytoplasm; FSP1, ferroptosis suppressor protein 1; CoQ, Coenzyme Q; DHO, dihydrolactate; mito, mitochondrial; OA, orotate; DAP, dihydroxyacetone phosphate; G3P, glycerol; PARL, presenilin associated rhomboid like; ESCRT-III, endosomal sorting complexes required for transport- III; CHMP5, chromatin-modifying protein 5; CHMP6, chromatin-modifying protein 6.

Lastly, a recent study has revealed the role of presenilin-associated rhomboid-like (PARL)-dependent STAR-related lipid transfer domain containing 7 (STARD7) in mitochondrial defense against ferroptosis. After cleavage and processing by the protease PARL, STARD7 can be localized in both mitochondria and the cytoplasm. These two forms of STARD7 have distinct functions: mitochondrial STARD7 facilitates CoQ synthesis, while cytoplasmic STARD7 transfers CoQ from mitochondria to the plasma membrane. The study also demonstrated a correlation between PARL or STARD7 expression and cancer cell resistance to the ferroptosis inhibitor protein GPX4, suggesting that targeting PARL or STARD7 could be a potential strategy to disrupt ferroptosis and provide novel approaches for anticancer therapies [140]. Figure 2 provides a visual summary of the mitochondrial defense mechanisms against ferroptosis.

### 3.8. Mitophagy and Ferroptosis

Mitochondria play a vital role in producing ATP for cellular energy, but they are susceptible to damage, leading to decreased ATP production and increased ROS generation. To maintain mitochondrial health, damaged mitochondria are eliminated through mitophagy, while new and healthy mitochondria are generated through mitochondrial biogenesis. Interestingly, there is a dynamic interplay between ferroptosis, a form of cell death, and mitophagy, which affects crucial mitochondrial processes. Studies have revealed that protein O-GlcNAcylation, an important post-translational modification linked to glucose metabolism, plays a role in regulating this interplay. It has been reported that decreasing O-GlcNAcylation promotes mitophagy, releasing iron from mitochondria and increasing the availability of labile iron, which can participate in the process of ferroptosis [150]. Autophagy, regulated by nuclear receptor coactivator 4 (NCOA4), selectively degrades ferritin through lysosomes when iron levels are high, leading to an elevation in intracellular free iron (Figure 1). Reducing ferritin O-GlcNAcylation enhances the interaction between ferritin and NCOA4, promoting ferritinophagy. Additionally, myoferlin targeting mediated by WJ460 has been shown to induce mitophagy, ROS accumulation, lipid peroxidation, and apoptosis-independent cell death [151]. Moreover, erastin inhibits O-GlcNAcylation, leading to increased GSH synthesis and the induction of ferroptosis in liver cancer cells [152]. These findings establish a previously uninvestigated connection between mitophagy and ferroptosis, shedding light on a new stage in the cell death process.

## 4. Mitochondria-mediated Ferroptosis in Relevant Diseases

Maintaining the normal morphology and physiological function of mitochondria is essential for supporting normal physiological metabolism and cellular development. Any alterations to the structure and function of mitochondria can contribute to the development of various diseases.

### 4.1. Mitochondria-mediated Ferroptosis in Cancer

Ferroptosis, a form of cell death induced by oxidative stress, is closely associated with cell metabolism. Tumors, characterized by their active metabolism, high ROS levels, and elevated iron supply, are more susceptible to ferroptosis [153, 154]. The erratic proliferation of cancer cells and the frequent occurrence of oxidation reactions within the cell contribute to the heightened tendency of tumors to undergo ferroptosis.

Certain subsets of tumor cells exhibit resistance to conventional treatments but have a higher propensity to undergo ferroptosis [155, 156]. Pancreatic ductal adenocarcinoma (PDAC) has an extremely low 5-year survival rate [157] and is resistant to many cancer therapies, making it the seventh leading cause of cancer-related deaths globally. Reports suggest that sensitizing PDAC cells to ferroptosis in vitro and in tumor xenograft studies [158, 159] by inhibiting system Xc- with erastin or through gene ablation offers a promising treatment option for this malignancy. Further research has revealed numerous cancer-related substances and signaling mechanisms that regulate ferroptosis. Mitochondria play a significant role in both ferroptosis and cancer evasion. In certain cases, cancer cells can evade the immune system through metabolic adaptability [160]. Recent discoveries linking metabolic reprogramming to ferroptosis sensitivity have expanded the possibilities for treating drug-resistant tumors, including those resistant to radiotherapy [161]. Manipulating mitochondrial metabolic pathways to reshape the tumor microenvironment may potentially induce ferroptosis and suppress tumor cell growth. The metabolic reprogramming in tumor cells is largely dependent on mitochondria. Cancer cells often shift their metabolism from glycolysis to mitochondrial oxidative phosphorylation (OXPHOS), rendering them vulnerable to GSH depletion and ferroptosis [162]. GLN breakdown provides bioenergy for most cancer cells [163]. Since GLN promotes ferroptosis, blocking its degradation could offer a novel strategy for cancer treatment.

**Figure 3.**
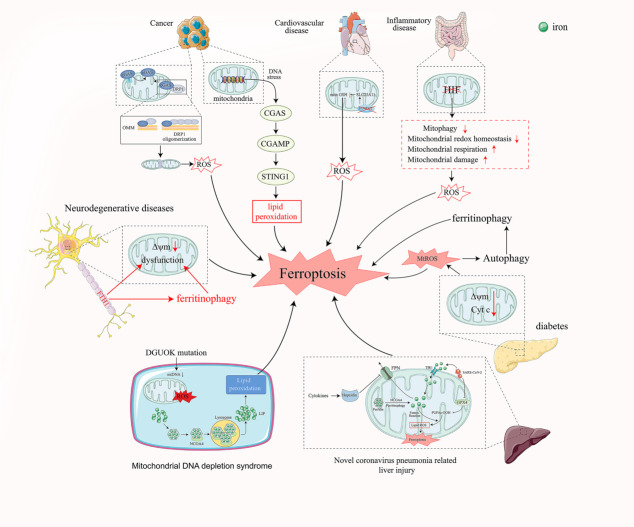
**Mitochondrial-mediated ferroptosis and its implication in diseases**. Cancer: mtDNA stress in tumor cells affects lipid metabolism and ferroptosis via the cGAS- STING pathway, with cGAS also promoting DRP1 oligomerization, affecting ROS production, and inducing ferroptosis. Cardiovascular diseases: FUNDC2 interacts with SLC25A11 to inhibit ferroptosis. Inflammatory disease: HIF in mitochondria affects a range of mitochondrial functions, which in turn affects ferroptosis. Neurodegenerative disease: FTH1 promotes ferroptosis by affecting mitochondrial function through ferritinophagy. Mitochondrial DNA depletion syndrome: Mutations in the DGUOK gene can lead to mitochondrial dysfunction and mtDNA damage, further causing ferroptosis. Novel coronavirus pneumonia: a viral liver infection resulting in iron transfer to cells via TfR1, inhibiting FPN, preventing iron flow out of cells and affecting intracellular iron levels. Additionally, SARS-CoV-2 reduces GPX4 expression, affecting intracellular ROS production and ultimately inducing ferroptosis. Diabetes: Ferroptosis of pancreatic beta cells emergency via the ROS-autophagy-lysosome pathway. Abbreviation used: TfR1, Transferrin Receptor 1; ROS, ROS; FPN, ferroportin; HIF, Hypoxia-inducible factor; cGAS, cyclic GMP-AMP synthase; STING1, stimulator of interferon response cGAMP interactor 1; FUNDC2, FUN14 domain-containing 2; mitoGSH, mitochondrial glutathione; DRP1, dynamin-related protein 1; DGUOK, deoxyguanosine kinase; NCOA4, nuclear receptor coactivator 4; OMM, outer mitochondrial membrane; SLC25A11, solute carrier family 25 member 11; cGAMP, cyclic GMP-AMP.

Research on mtDNA mutations in cancer pathology [164, 165] has emphasized the importance of mtDNA function in human health and disease. Zalcitabine, an antiviral medication, has been shown to induce ferroptosis and suppress the proliferation of human pancreatic cancer cells by reducing mtDNA copy number, impairing respiration, decreasing oxygen utilization, and inhibiting ATP production. The induction of mtDNA stress by Zalcitabine activates the cGAS-STING1 pathway, leading to autophagy-dependent ferroptosis in pancreatic cancer cells [166]. STING1, a transmembrane protein localized on the endoplasmic reticulum membrane, regulates ferroptosis in PDAC by acting as a positive regulator of autophagic flux [167, 168]. In hepatocellular carcinoma (HCC) cells, it has been demonstrated that the cGAS-STING pathway is not involved in regulating the mitochondrial fission regulator DRP1. Instead, cGAS promotes DRP1 oligomerization on the OMM to modulate mitochondrial fission, reduce ROS production, and initiate ferroptosis (Figure 3) [169]. These findings provide insights into the role of cGAS in cancer and suggest mitochondria-mediated ferroptosis as a potential therapeutic strategy for targeting tumor cells. Tumor cells prioritize the health of their mitochondria due to their higher mitochondrial content than normal cells. The involvement of mitochondria in ferroptosis and other mitochondrial morphological and functional alterations raises important questions about current cancer treatments.

Overall, accumulating evidence suggests that targeting mitochondria and mitochondria-associated signaling pathways holds promise as an anticancer therapeutic strategy.

### 4.2. Mitochondria-mediated Ferroptosis in Cardiovascular Diseases

The cardiovascular diseases with the highest global mortality rates are myocardial infarction and heart failure[170]. These conditions are primarily caused by functional impairment or cell death of terminally differentiated cardiomyocytes [171, 172]. While apoptosis has been extensively studied, emerging evidence suggests that ferroptosis may also contribute to cardiomyocyte death in cardiac ischemia/reperfusion (I/R) injury [13]. Moreover, the mechanisms underlying cardiomyocyte ferroptosis involve mitochondria-dependent pathways [13, 173], highlighting the significance of mitochondria in maintaining normal cardiomyocyte function [174, 175].

In DOX-induced cardiac injury, the inducible heme oxygenase Hmox1 is upregulated by the nuclear translocation of Nrf2, leading to heme degradation and increased free iron levels. This elevation in free iron drives ferroptosis and contributes to heart failure in cardiomyocytes exposed to DOX or DOX-induced lipid peroxidation. Inhibiting cardiac cell ferroptosis by targeting mitochondrial-mediated pathways can significantly reduce DOX-induced cardiomyopathy [13]. Additionally, in the context of diabetes, it has been demonstrated that Isorhapontigenin (ISO) can inhibit ferroptosis through the PRDX2-MFN2-ACSL4 pathways. ISO treatment upregulates PRDX2, affects MFN2, and inhibits mitoACSL4 mitochondrial translocation, leading to improved mitochondrial dynamics and functional inhibition of ferroptosis [176]. These results suggest that mitochondrial oxidative damage plays a primary role in ferroptosis-related cardiac damage. Therefore, reducing iron accumulation in mitochondria and lipid peroxidation during acute and chronic cardiac I/R may have cardioprotective effects. Selectively inhibiting ferroptosis in cardiomyocytes could be a potential therapeutic approach for managing DOX-induced cardiac injury or other conditions where anthracyclines are used as anticancer agents, such as breast cancer, leukemia, and other malignancies [177], without compromising the drug's anticancer properties.

Despite ferroptosis being a well-known contributor to cell death in cardiac I/R injury[13], there is limited research on compounds that can resist ferroptosis [178]. A recent discovery has identified a new ferroptosis inhibitor called lip-1. In experiments using isolated mouse hearts, post-ischemic treatment with lip-1 was found to reduce the size of myocardial infarcts and protect the structural integrity and function of mitochondria, resulting in cardiac protection. The cardioprotective effects of lip-1 were mediated by a decrease in VDAC1 levels and oligomerization but not VDAC2/3. Additionally, lip-1 treatment reversed the decrease in the antioxidant GPX4 induced by I/R stress and reduced mitochondrial ROS production. Consequently, it is plausible that lip-1 induces cardioprotective effects against I/R injury by focusing on ferroptosis. Thus, these findings support the hypothesis that mitochondrial ROS production plays a crucial role in ferroptosis [179, 180], offering a promising therapeutic option for the treatment of cardiovascular diseases by targeting mitochondrial ROS production to inhibit ferroptosis. Further evidence has demonstrated the impact of mitochondrial ROS on ferroptosis in the presence of specific inhibitors, providing additional confirmation of the involvement of mitochondrial damage in ferroptosis [31, 181].

Overall, existing literature supports the notion that mitochondria or their signaling pathways directly suppress ferroptosis in cardiomyocytes, which may in turn promote the development of new therapies for managing drug-resistant heart diseases and tumors with cardiotoxicity resulting from drug side effects.

### 4.3. Mitochondria-mediated Ferroptosis in Inflammatory Disease

Ferroptosis, a detrimental form of cell death, is triggered by lipid peroxidation induced by excessive ROS levels [182, 183]. Numerous investigations have linked iron and ROS to the progression of ulcerative colitis[184-186]. Mitochondria play a crucial role in generating intracellular ROS and regulating intracellular iron levels to initiate ferroptosis. Emerging evidence suggests that ferroptosis may exert a protective role in inflammation, and various drugs have demonstrated anti-inflammatory and ferroptosis-inhibitory effects in animal models. Thus, it is plausible to consider that mitochondria-mediated ferroptosis may contribute to the pathogenesis of inflammatory disorders.

Septicemia-related acute kidney injury (AKI) is a condition associated with significant morbidity and mortality [187] and is characterized by a rapid rate of O2 depletion in the renal mitochondria[188]. This suggests an abnormality in certain oxygen-consuming responses within the mitochondria, which may be triggered by even a modest disruption in the mitochondrial signaling pathways, can lead to a wide range of pathogenic reactions in renal cells. Hypoxia-inducible factor-1 (HIF-1), a crucial regulator of oxygen homeostasis, has been implicated in protecting against ischemia-related kidney damage [189]. Additional evidence[190] shows that HIF has a protective effect on renal IRI, which is mediated by complicated processes, including regulating ROS generation and inflammation. However, only a few studies have investigated the link between HIF activation and ferroptosis. The hypothesized roles of HIF in AKI include the prevention of ferroptosis and the decrease in mitochondrial respiration. The TCA cycle and ETC activity in the mitochondria are known to initiate ferroptosis under specific conditions. Figure 3 [191] illustrates the complex chain of events showing that HIF can suppress the TCA cycle and ETC activity. The mitochondrial uncoupler CCCP has been shown to be effective in mitigating the effects of hypoxia on the kidneys [192]. However, in cases where mitochondria are excessively active, enabling numerous chemical reactions to occur rapidly and efficiently, it remains uncertain which specific compounds are associated with ferroptosis induction in AKI.

Upregulation of NRF2 and downstream proteins such as HO-1 and GPX4 [14] has been associated with HIF's ability to suppress ferroptosis. HIF promotes the synthesis of GSH, a major antioxidant that protects against ferroptosis, while also maintaining redox homeostasis and reducing ferroptosis [193]. By maintaining mitochondrial redox homeostasis, HIF may protect against ferroptosis in AKI. The transcription of VDAC1 is regulated by OMM-linked HIF-1, which is known to protect mitochondria[194, 195]. It is now recognized that mitochondrial dysfunction can trigger inflammatory responses through specific signaling cascades. In the presence of viral caspase inhibitors that limit apoptotic caspase activation [196], mtDNA can activate potent inflammatory responses via the cGAS-STING1 pathway upon MPT or MOMP. Additionally, the release of mtDNA and ROS resulting from mitochondrial dysfunction can stimulate the production of interleukin (IL)-1 and IL-18, whereby mtDNA and ROS act as primary DAMPs that trigger inflammatory responses affecting RCD [197] via intricate networks that intersect with molecular mechanisms at distinct nodes. As previously observed, the link between inflammation and ferroptosis is strengthened by the involvement of mtDNA and mitochondrial ROS. Considering the involvement of mitochondria in both ferroptosis and inflammation, further investigation into mitochondrial-mediated processes and mitochondrial signaling is warranted.

### 4.4. Mitochondria-mediated Ferroptosis in Neuro-deenerative Diseases

Progressive loss of neuronal, motor, and/or cognitive function is a hallmark of neurodegenerative disorders (NDD), but little is known about the underlying pathogenic mechanism. NDD can lead to the gradual and permanent death of neurons in some parts of the brain and spinal cord[198]. In the brain, oxidative damage primarily manifests as lipid peroxidation due to the abundance of lipids rich in polyunsaturated fatty acids. Lipid peroxidation is believed to play a significant role in the development of NDD [199]. The involvement of oxidative stress as a mediator between neuronal dysfunction and death in age-related NDDs is widely recognized. Researchers investigating various neurodegenerative paradigms have identified uncontrolled intracellular oxidative stress as a constant source of neuronal death signals. Overall, ferroptosis has emerged as one of the key mechanisms through which these diseases can propagate [136].

Recent research has provided direct evidence linking oxidative death and neurodegeneration by demonstrating that severe mitochondrial damage leads to the loss of mitochondrial integrity and function, resulting in the accumulation of iron and ROS in specific brain regions. Abnormalities in mitochondrial iron metabolism, a process associated with ferroptosis, have been implicated in many neurodegenerative disorders, including Alzheimer's disease (AD) and Parkinson's disease (PD)[200]. Studies have revealed significant iron accumulation in various regions of the brain affected by AD [201], which is a progressive and irreversible neurodegenerative disorder characterized by impaired cognition. The buildup of iron is associated with both the amount and rate of accumulation of β-amyloid plaques, which are closely associated with cognitive impairment in AD patients [202, 203]. Recent studies have provided evidence of ferroptosis in AD, including dysregulation of iron homeostasis and lipid peroxidation [204]. FtMt is expressed only in highly oxygen-depleted tissues, such as testicular cells and mitochondria of central nervous system cells[205]. Previous research has shown that FtMt regulates cellular iron metabolism, reduces cytoplasmic iron accumulation, and protects against erastin-induced ferroptosis in neuroblastoma SH-SY5Y cells[102]. Furthermore, the impairment of mitochondrial metabolism due to the suppression of mitochondrial respiration and ATP synthesis, the inhibition of cellular antioxidant activity, and the induction of ferroptosis in human astrocytes[206] has been shown to contribute to increased NOX4 levels, which in turn promotes oxidative stress. The PD-specific iron-storage protein FTH1 has been shown to play a role in maintaining normal cellular physiological function, mitochondrial function integrity and mitochondrial potential by binding to NCOA4 and recruiting microtubule-associated protein light chain 3, which in turn participates in ferritinophagy to further influence mitochondrial potential. In 6-OHDA-treated PC-12 cell models of PD, chloroquine and bafilomycin A1, which are inhibitors of ferritinophagy, were found to reduce ferritin degradation and ferroptosis[207]. In addition, the observed mitochondrial membrane potential reduction, ATP production decrease and mitochondrial ROS increase triggering ferroptosis [200] in PC-12 cells further support the involvement of mitochondria in neurodegeneration-related iron apoptotic cell death.

Taken together, there is strong evidence that mitochondria-mediated ferroptosis plays a role in NDD, and treatments that target its mechanisms, especially via iron chelation, were discovered to be therapeutically effective in clinical trials, which may pave the way for novel anti-ferroptosis treatment strategies in patients with NDD[208, 209]. Thus, treating diseases via mitochondria as an intervention in oxidative cell death holds significant promise. However, to fully harness the potential of mitochondria in disease treatment, it is imperative to conduct further research into the molecular functions of mitochondria in the neurological system.

### 4.5. Mitochondria-mediated Ferroptosis in mtDNA Depletion Syndrome

mtDNA depletion syndrome (MDS) is a severe genetic disorder characterized by a significant reduction in mtDNA levels in affected organs and tissues [210]. Despite this, there have been limited studies exploring the association between ferroptosis and MDS. Mitochondrial dysfunction caused by mutations in the DGUOK kinase results in decreased ATP production, increased ROS levels, and depletion of the antioxidant GSH [211]. Simultaneously, the release of LIP during ferritin degradation in NCOA4-dependent lysosomes can lead to lipid peroxidation and ferroptosis [212]. These findings establish a connection between DGUOK mutations in MDS and iron overload-induced ferroptosis. It has been reported that the liver, particularly in DGUOK mutated patients with mtDNA deletion and respiratory dysfunction, is particularly susceptible to ferroptosis induced by iron overload (Figure 3) [213]. Therefore, the treatment of MDS requires a tailored approach that considers the specific manifestations and needs of each patient.

### 4.6. Mitochondria-mediated Ferroptosis in Novel Coronavirus Pneumonia

The rapid disease progression observed in COVID-19 patients has been associated with an exaggerated inflammatory response, which is influenced by disturbances in iron metabolism and reduced oxidative stress [214]. Studies have indicated that SARS-CoV-2 infection can enhance iron uptake through the transferrin receptor 1 (TfR1) in liver cells [215]. SARS-CoV-2 virus requires iron to replicate. Subsequently, NCOA4 facilitates the release of ferric iron by eliminating excess ferritin, and STEAP family member 3 converts Fe3+ to Fe2+. The inflammatory response triggered by SARS-CoV-2 infection activates the hepcidin pathway, inhibiting iron export by FPN and promoting the onset of ferroptosis. Additionally, interleukin 6 (IL-6) promotes the synthesis of ferritin and hepcidin, further impeding iron export and facilitating the accumulation of excess iron in mitochondria through mitochondrial ferritin, where it contributes to lipid peroxidation, a process that in turn affects the structural integrity of the mitochondrial membrane and leads to ferroptosis[216]. The accumulation of intracellular iron triggers the Fenton reaction and excessive lipid ROS, resulting in liver damage associated with COVID-19, known as ferroptosis. SARS-CoV-2 infection has been shown to downregulate GPX4 expression, increase lipid ROS production, accelerate the Fenton reaction under conditions of iron overload, and promote ferroptosis (Fig. 3). Therefore, investigating the role of mitochondria-mediated ferroptosis may provide new insights into the pathogenesis of COVID-19.

### 4.7. Mitochondria-mediated Ferroptosis in Diabetes

Numerous studies have investigated the impact of diabetes on apoptosis, necrosis, and autophagy[217, 218]. However, the role of mitochondrial-mediated ferroptosis in the pathogenesis of diabetes has received less attention. Severe dysfunction of the pancreas is strongly associated with type 2 diabetes (T2D), primarily due to mitochondrial failure, a key mechanism of ferroptosis that is exacerbated in diabetes. The loss of pancreatic beta cells contributes to pancreatic dysfunction [219, 220]. Exposure to arsenic significantly increases the risk of developing T2D and pancreatic dysfunction. In MIN6 cells, arsenic induces autophagy and ferritinophagy by reducing cytochrome C levels, altering mitochondrial membrane potential, and decreasing ROS generation. Arsenic disrupts intracellular iron homeostasis, leading to ferroptosis. The detrimental effects of arsenic on T2D and pancreatic dysfunction can be mitigated by using the MtROS scavenger Mito-Tempo, the autophagy inhibitor chloroquine, and Fer-1. These findings provide evidence for the connection between T2D and the mitochondrial ROS-autophagy-lysosome pathway [221]. Thus, the pathophysiology of mitochondrial ferroptosis in diabetes is complex and involves multiple processes. Enhancing our understanding of these mechanisms may enable the development of more effective therapeutic strategies, and combining inhibitors targeting multiple pathways may offer a promising approach to maximize therapeutic efficacy in treating diabetes.

## 5. Strategies to Target Mitochondria-mediated Ferroptosis for Therapy

Targeting mitochondria-mediated ferroptosis as a therapeutic strategy is an emerging treatment strategy with multiple pathways. One approach is to reduce iron concentrations by modulating the homeostasis of iron ion levels within mitochondria. This can be achieved by utilizing drugs that regulate the uptake and transport of iron ions in mitochondria. Another strategy is to target oxidative damage and ferroptosis resulting from elevated iron ion concentrations in mitochondria. Examples include the use of inhibitors of iron transporters and ferritin to treat genetic diseases. In the case of mitochondrial membrane protein-associated neurodegeneration (MPAN), a rare genetic disorder, the antioxidant deferoxamine (DFO) has been shown to inhibit ferroptosis by alleviating oxidative stress and mitochondrial defects. MPAN fibroblast cells are highly sensitive to ferroptosis induction by agents like erastin or RSL 3, but pretreatment with DFO can effectively reverse this sensitivity. Iron chelators such as DFO and Deferiprone (DFP) have been extensively employed in treating hypoxic injury and certain neurodegenerative diseases. Excessive iron accumulation in the mitochondria has been identified in Friedreich's ataxia-associated ferroptosis, resulting in heightened mitochondrial oxidation. Notably, treatment with the mitochondria-targeted antioxidant XJB-5-131 has shown effective inhibition of ferroptosis [222].

Iron chelators have shown great potential in therapeutic applications, particularly in reducing total iron levels. DFO, known for its high affinity for Fe3+, can effectively lower iron levels. By interfering with ISC and heme synthesis, MitoDFO regulates mitochondrial iron levels, inhibits ferroptosis, and maintains systemic iron homeostasis[223]. MitoDFO regulates mitochondrial iron levels by interfering with ISC and heme synthesis, which inhibits ferroptosis without affecting systemic iron levels. MitoDFO can also inhibit ROS production, halt mitochondrial respiration, and induce mitophagy. DFO has been shown to inhibit the proliferation of leukemic cells [224]. The long-term use of DFO has been associated with peripheral nervous system abnormalities and other possible adverse effects in humans. Deferasirox (DFX) is an enhanced iron chelator that has gained popularity in recent years due to its longer elimination half-life compared to DFO [225, 226]. In mitochondria, inhibition of the ferroptosis pathway could conceivably be targeted. Pharmaceutical drugs can prevent mitochondrial damage by obstructing the initial phase of the intramitochondrial ferroptosis pathway. Gallium maltolate (GaM) promotes ferroptosis in triple-negative breast cancer (TNBC) by altering mitochondria's morphology and membrane potential.

Collectively, targeting mitochondrial-mediated ferroptosis through modulation of iron ion homeostasis, antioxidant interventions, and inhibition of the intra-mitochondrial ferroptosis pathway have shown promise in developing effective therapeutic strategies. However, it is important to acknowledge that ferroptosis-targeting drugs can be toxic, affecting normal cells and potentially causing side effects. Therefore, thorough investigation, monitoring, and addressing of potential adverse effects and safety concerns are crucial in the clinical application of these drugs.

## 6. Conclusion Remarks and Future Perspectives

Ferroptosis, a recently discovered form of RCD, is characterized by GSH depletion, GPX4 inactivation, and accumulation of lipid peroxidation. It is an emerging field of research with significant implications. Mitochondria, the main energy producers in the cell, play a central role in various metabolic processes. They are also involved in cellular signaling and participate in diverse signaling pathways. Targeting ferroptosis, a mitochondria-mediated cell death process, holds promise due to its profound metabolic relevance. Uncovering mitochondrial markers associated with ferroptosis will greatly enhance our understanding of this phenomenon. The distinct metabolic profile of cancer cells makes ferroptosis activation a potential strategy to hinder cancer growth. Dysregulation of ferroptosis can contribute to cellular degeneration and tissue damage in various human diseases, particularly those affecting the cardiovascular system. Despite the promising therapeutic prospects of targeting ferroptosis, there are still challenges that need to be addressed.

Currently, the specific signaling molecules associated with mitochondria-mediated ferroptosis remain unidentified, and it seems that lipid peroxidation, induced by various triggers, is the primary driver of this process. The existence of additional unidentified effector molecules, in conjunction with lipid peroxidation, and their roles in initiating ferroptosis in different cellular contexts are still unclear. Growing evidence suggests a connection between mitochondrial dysfunction and ferroptosis. However, due to the dynamic nature of mitochondria, it remains uncertain whether mitochondrial dysfunction alone is sufficient to trigger ferroptosis and whether mitochondrial function, division, or fusion impact ferroptosis under specific conditions. Studies have demonstrated that dysfunction of MAMs can induce ferroptosis, but the involvement of other organelles in ferroptosis and the interplay between mitochondrial signaling and other organelles remain poorly understood. Further research is needed to unravel the intricate relationship between MAMs, mitochondria, and ferroptosis.

Mitochondria-mediated ferroptosis is an emerging concept with promising potential for treating various diseases. While this review has provided examples of its application in liver-associated digestive disorders and chronic obstructive pulmonary disease, there are numerous other conditions in which mitochondria-driven ferroptosis may occur. It is important to emphasize that a comprehensive summary should encompass innovative treatment approaches targeting mitochondria-mediated ferroptosis.

While targeting mitochondria-mediated ferroptosis holds promise for therapeutic interventions, there are several limitations and challenges that must be addressed. One major limitation is the lack of drug specificity, as many pharmaceuticals targeting ferroptosis may also affect other forms of cell death. This lack of specificity can lead to potential harm and side effects in normal cells. Moreover, the effectiveness of drug delivery to mitochondria is hindered by the cell membrane barrier, which limits the direct targeting of mitochondria-mediated ferroptosis. Innovative drug delivery methods, such as the use of nanomaterials, should be explored to enhance targeted delivery to mitochondria. Another challenge is the need for improved detection techniques specific to ferroptosis. As a relatively new area of research compared to other cell death mechanisms, the development of reliable and sensitive methods for detecting and monitoring ferroptosis is essential for its translation into clinical practice. Overall, while there is promise in targeting mitochondria-mediated ferroptosis, further investigation is required to overcome the limitations and challenges associated with its clinical application.

For clinical treatments to be successful, certain issues must be addressed. First, it is important to determine the most suitable patients, cancer types for ferroptosis-targeted treatment and whether the treatment or therapeutic agent could be detrimental to other organs. Additionally, test results should be evaluated to determine the optimal treatment approach for a patient, based on which a suitable treatment strategy should be formulated. As the tumor microenvironment is a crucial factor in cancer progression and treatment response, understanding the intricate relationship between the tumor microenvironment, tumor metabolism and ferroptosis is essential. It is unclear under what conditions mitochondria-mediated ferroptosis may have a pro- or anti-inflammatory effect, as the effect of this process on the inflammatory response is not definite. By addressing these difficulties, we may better understand mitochondria-mediated ferroptosis and improve its application in clinical settings.
